# A review of new challenges and prospects for malaria elimination in Mutare and Mutasa Districts, Zimbabwe

**DOI:** 10.1186/s12936-016-1415-2

**Published:** 2016-07-13

**Authors:** Shadreck Sande, Moses Zimba, Peter Chinwada, Hieronymo Takundwa Masendu, Joseph Mberikunshe, Aramu Makuwaza

**Affiliations:** Department of Biological Science, University of Zimbabwe, Harare, Zimbabwe; ABT Associates Inc., 1 Erskine Road, Mt Pleasant, Harare, Zimbabwe; Ministry of Health and Child Care, National Malaria Control Programme, Harare, Zimbabwe; National Institute of Health Research, Causeway, Harare, Zimbabwe

**Keywords:** Malaria, New challenges, Malaria vectors, Malaria elimination

## Abstract

This review outlines and discusses the new challenges in malaria control and prospects for its elimination in Mutare and Mutasa Districts, Zimbabwe. The burden of malaria has declined significantly over the past 5 years in most regions in Zimbabwe, including Mutare and Mutasa Districts. The nationwide malaria reduction has been primarily linked to scaled-up vector control interventions and early diagnosis and treatment with effective anti-malarial medicines. The successes recorded have prompted Zimbabwe’s National Malaria Control Programme to commit to a global health agenda of eliminating malaria in all districts in the country. However, despite the decline in malaria burden in Mutare and Mutasa Districts, there is clear evidence of new challenges, including changes in vector behaviour, resistance to insecticides and anti-malarial medicines, invasion of new areas by vectors, vectors in various combination of sympatry, changes in vector proportions, outdoor malaria transmission, climate change and lack of meticulousness of spray operators. These new challenges are likely to retard the shift from malaria control to elimination in Mutare and Mutasa Districts.

## Background

Following the aborted Global Malaria Eradication campaign in the 1960–1970s, malaria received little international attention over the subsequent years until recently [1]. After the launch of the Roll Back Malaria (RBM) programme in 1998, most countries with endemic malaria, especially in Africa, made substantial progress in their malaria control interventions. Currently, it appears commitment has greatly improved, and partnerships exist to accelerate and sustain malaria control and elimination to achieve national, regional and global malaria targets and the malaria-related Millennium Development Goals (MGDs) [2]. Malaria elimination has been defined as permanent reduction to zero incidences of locally contracted cases [3]. The malaria target under MGD 6 (halting and beginning to reverse the incidence of malaria by 2015) has been met and 55 countries are on track to reduce their malaria burden by 75 % in line with the World Health Assembly’s target of 2015. Malaria mortality decreased by 47 % between 2000 and 2013 globally, and by 54 % in the World Health Organization (WHO) African region, with an increasing number of countries striving towards malaria elimination [4]. This progress is primarily attributed to scaled-up vector control interventions, especially indoor residual spraying (IRS) and long-lasting insecticidal nets (LLINs), as well as improved malaria diagnosis and effective treatment. Implementation of malaria control strategies in Zimbabwe has not been disturbed by any political situation during the past 5 years.

In Zimbabwe, vector control is a central, critical component of all malaria control strategies and the use of IRS and LLINs has increased immensely over the past decade as part of an effort towards universal coverage of all populations at risk of contracting the disease. If a universal coverage and greater than 80 % use of IRS and LLINs by populations at risk of malaria are attained, consolidated and maintained, malaria transmission will be significantly reduced [2]. Over the years, new challenges have emerged, complicating the goal of controlling and eliminating malaria. Despite Zimbabwe being a member of the Malaria Elimination 8 (E8) countries in the Southern Africa, the new threats and prospects for a successful shift from malaria control to elimination in Mutare and Mutasa Districts are not well understood. The article reviews work on malaria parasites, vector species composition, insecticide resistance and responses in vector mosquitoes following prolonged use of IRS and LLINs. In this review, the aim was to identify and describe common new challenges and prospects for malaria elimination in Mutare and Mutasa Districts, Zimbabwe, where substantial and constant strides have been made towards control.

## Selected districts and data collection

**Fig. 1:**
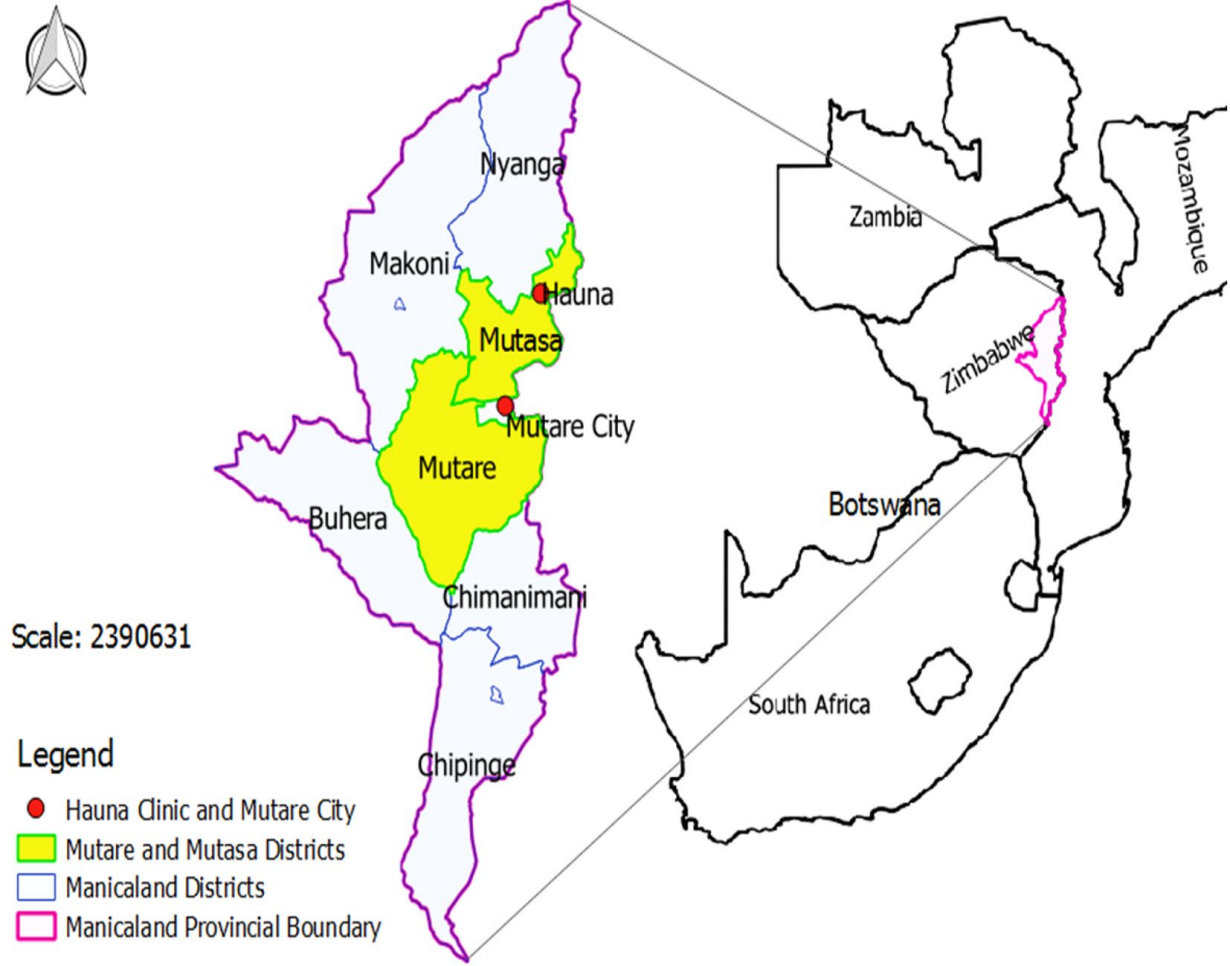
Map showing Mutare and Mutasa study sites, Zimbabwe

Mutare (19°39′S, 32°27′E; elevation 1063 m) and Mutasa (18°29′S, 32°50′E; elevation 912 m) Districts in Manical and Province (Fig. 1), Zimbabwe, are selected for review as they are among some of the few areas for which historical entomological data and related information is available. The two districts are neighbouring areas situated to the eastern part of Zimbabwe, with their district administrative headquarters being separated by a distance of about 90 km. The intensity of malaria transmission in the two districts differs considerably, with Mutasa District always in the lead. In the two districts, 95 % of all malaria cases are caused by *Plasmodium falciparum* [5] and primarily transmitted by *Anopheles funestus* sensu stricto [6]. The disease is seasonal, but prone to sporadic epidemics, and is considered a public health problem in the two districts. Indoor residual spraying and LLINs are the major vector control strategies employed to combat malaria. In 2014, IRS protected over 80 % of the population at risk of malaria in the two districts (Mberikunashe, unpublished data). However, the proportion of the population protected by use of mosquito nets is not clear.

Information on mosquito vector control, behaviour, and epidemiology in Zimbabwe is available from work by various researchers as well as unpublished data from sources such as the National Malaria Control Programme (NMCP), National Institute of Health Research (NIHR), national archives and academic institutions. Work by Mpofu [7], Taylor and Mutambu [8], Masendu et al. [9] and Sande et al. [6], reported extensively on malaria species composition and relative abundance in various regions of Zimbabwe. Leeson [10], Alves and Blair [11], Mabaso et al. [12] and Munhenga [13] documented the history of vector control through use of IRS as far back as the 1940s. Masendu [14], Dandalo [15] and Sande et al. [16, 17] reported on the biting and resting behaviour of vector mosquitoes from 1996 to 2016 in various parts of Zimbabwe. Elsewhere in Africa, some changes in vector behaviour including resting and biting have been attributed to prolonged use of IRS and LLINs [18].

## Malaria situation prior to the house-spraying and mosquito net era

Prior to the implementation of IRS and/or LLINs, endemicity of malaria in Zimbabwe was shown to be markedly influenced by altitude, varying from hyperendemic in the low attitude areas (elevation less than 700 m) to hypoendemic or completely absent on the central watershed (elevation more than 1200 m) [8, 10, 11]. Malaria transmission was intense, yet clearly seasonal, peaking from February to April, and the geographical distribution was more extensive, with sporadic epidemics in some areas [10, 11], including Mutare and Mutasa Districts. Random malariometric surveys, especially parasite rates were carried out as pre-control strategies in selected districts [19].

## Malaria situation after the introduction of house-spraying and mosquito nets

Over the last decade, Zimbabwe has recorded a steady annual decline in malaria morbidity, from an annual incidence of 153 cases per 1000 populations in 2004, to 29 cases per 1000 populations by the end of 2013. Malaria deaths decreased from approximately 3000 in the early 2000s to about 300 people per annum in recent years [20]. A clear reduction in malaria burden was observed in the southern and central parts of Zimbabwe, with Matabeleland South Province recording malaria cases of less than 1 per 1000 populations in 2012 [20]. From late 2012, the NMCP upgraded Matabeleland South Province from implementing malaria control activities to pre-elimination.

**Fig. 2:**
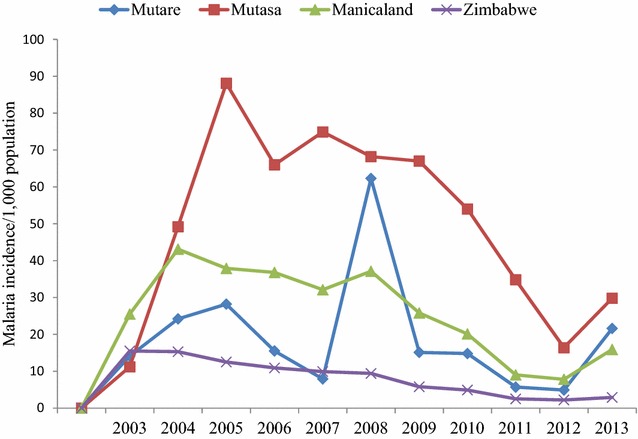
Malaria incidence per 1000 population in Mutare and Mutasa Districts from 2003–2013

The reduction in malaria burden in Zimbabwe has played a pivotal role in giving confidence to politicians, policy makers, health workers and funding agencies to keeping malaria elimination high in the national agendas. All gains have coincided with widespread adoption of various malaria control strategies, especially IRS, LLINs, and early diagnosis and effective treatment. It appears the new challenge is that most of the milestones achieved in malaria control over the years are unevenly distributed and breakable in Zimbabwe, especially in Mutare and Mutasa Districts. However, from 2003 to 2013, the malaria incidence (Fig. 2), though declining, remained relatively high in Mutare (19.5 %, range 4.9–62.3 %) and in Mutasa (50.9 %, range 11.2–88.1 %) (Zimbabwe District Health Information System 2 [ZDHIS 2], unpublished data). However, malaria control interventions were enhanced from 2009 (Mberikunashe, unpublished data). Prior and following enhanced malaria control interventions, malaria incidence rates were 21.7 % (range 7.9–62.3 %) and 12.4 % (range 4.9–21.6 %) respectively in Mutare, and 59.1 % (range 49.2–88.1 %) and 29.2 % (range 11.2–54.0 %) respectively in Mutasa (Zimbabwe District Health Information System 2 [ZDHIS 2], unpublished data).

## Status of malaria elimination

In 2009, a meeting was held by Ministers of Health of eight Southern African countries, the Malaria Elimination 8 (E8), in Windhoek, Namibia, to deliberate on the mechanisms and partnerships necessary for malaria elimination in their sub-region [21]. A subsequent E8 inaugural meeting was held in Maputo, Mozambique in 2010, which served as a forum for the Ministers of Health of the eight countries to coordinate efforts and assess progress made towards malaria elimination [21]. Four frontline countries (Botswana, Namibia, Swaziland and South Africa) for E8 were positioned to immediately move from malaria control to elimination, while the remaining four (Angola, Mozambique, Zambia and Zimbabwe) were expected to consolidate malaria control, supporting the frontline countries and preparing the transition to malaria elimination phase.

Resulting from the Maputo meeting, the malaria situation was assessed in all eight rural provinces of Zimbabwe following WHO guidelines [22]. The criterion for zonal classification into malaria programme phases and milestones on the path to malaria elimination was followed, with control and consolidation (slide positive rate <5 % in all fever cases), pre-elimination (<1 case/1000 population at risk per year), elimination (0 local acquired cases), and prevention of reintroduction (WHO certification, 3 years without local transmission) [22]. The first province in Zimbabwe to implement activities under malaria pre-elimination/elimination phase was Matabeleland South in 2013. Currently, Matabeleland North, Midlands and Mashonaland West Provinces have also been promoted to implement malaria pre-elimination/elimination activities in some districts with effect from 2015. The remaining four rural provinces (Masvingo, Mashonaland Central, Mashonaland East and Manicaland) are strongly expected to continue to implement activities in the control phase, but under tight surveillance for a possible move to pre-elimination and elimination.

## Parasite and vector species composition

The predominant malaria parasite species in Zimbabwe is *P. falciparum* which accounts for over 95 % of malaria cases in the country [5]. The other few malaria cases are caused by *Plasmodium malariae* and *Plasmodium ovale.* The most important vector species which transmit human malaria in Africa belong to members of the *An. gambiae* complex and the *Anopheles funestus* group. In Zimbabwe, Mpofu [7], Taylor and Mutambu [8] and Masendu et al. [9] confirmed the presence of four members of the *An. gambiae* complex: *An. gambiae s.s.* (hereafter referred to as *An. gambiae*), *Anopheles arabiensis*, *Anopheles merus* and *Anopheles quadriannulatus*. More recently, Sande et al. [6] reported the sympatric occurrence of *An. arabiensis* and *An. quadriannulatus* in Mutare and Mutasa Districts, Zimbabwe. Within the *An. gambiae* complex, *An. arabiensis* and *An. gambiae* are the major human malaria vectors in sub-Saharan Africa [23].

Previous studies on the *An. funestus* group by Evans and Leeson [24] in Zimbabwe, reported the presence of *An. funestus s.s.* (hereafter referred to as *An. funestus*), *Anopheles leesoni* and *Anopheles confusus*. Green and Hunt [25] reported *An. funestus*, *Anopheles parensis* and *Anopheles aruni* in sympatry in various parts of Zimbabwe. More recently, *An. funestus* and *An. leesoni* sibling species were detected in Mutare and Mutasa Districts [6, 26]. In the *An. funestus* group, *An. funestus* is the only member that is implicated as an important vector of malaria in sub-Saharan Africa [27].

From as far back as the early 1970s, *An. arabiensis* was noted to be the primary vector of malaria in Zimbabwe while *An. gambiae* and *An. funestus* are secondary vectors [8, 9]. A nationwide vector distribution survey in Zimbabwe in 2005 reported the presence of *An. funestus* only at Buffalo Ranch in Chiredzi District of Masvingo Province, in the southern region of the country [9]. The scarcity of *An. funestus* was attributed to its elimination following decades of IRS. Interestingly, a 2013–2014 study on vector species composition in Mutare and Mutasa Districts showed the resurgence of *An. funestus* in the two districts [6]. The study demonstrated the shift in dominance of *An. funestus* from a secondary to a primary vector (95.4 %), with *An. arabiensis* being relegated to a secondary vector (4.6 %) in the two districts. In the absence of recent species composition data from other parts of Zimbabwe, the resurgence of *An. funestus* in Mutare and Mutasa could be more widespread than previously thought.

The supremacy of *An. funestus* in Mutare and Mutasa Districts is a new challenge to malaria control and elimination, primarily because it is a more efficient vector than *An. arabiensis* [28, 29]. Additionally, *An. funestus* is fairly difficult to collect in its larval stage and its adaptability to field insectary and laboratory conditions is poor, leading to inconsistent entomological studies using it. Regular entomological monitoring of vector species is of paramount importance to malaria control and elimination in any setting. The predominantly indoor resting and host-seeking traits of *An. funestus* reported by Pates and Curtis [18] in various parts of Africa and Sande et al. [16, 17] in Mutare and Mutasa set opportunities for its control using IRS or LLINs, with prospects of achieving the malaria elimination goal when combined with other effective malaria interventions.

## House-spraying and use of mosquito nets for malaria control

In Zimbabwe, IRS was started as a pilot study as far back as the 1940s using dichloro-diphenyl-trichloro-ethane (DDT) and then benzene hexachloride (BHC) [11, 12, 19], and is currently the mainstay of malaria vector control in the country. In 1986, following years of DDT use, deltamethrin was evaluated in Zimbabwe in experimental huts and the residual effect was found acceptable for malaria vector control [8]. Again in 1986, micro-encapsulated deltamethrin was tried under field conditions and recommended for widespread spraying in the country [8]. Later, in 1990, lambda-cyhalothrin was tested in a small community and the residual activity was found to be comparable to deltamethrin and suitable for nationwide use.

Since the1940s, residual spraying with DDT and more recently with pyrethroids has been the National Malaria Control Programme’s (NMCP) major vector control intervention with the aim of reducing malaria burden [30]. Over the past 5 years, implementation of IRS followed the WHO recommendation of achieving spray and population coverage of at least 80 %. The spray coverage and the proportion of population protected from 2009 to 2014 are shown on Table 1, with above 80 % spray and population protected coverage overall. This milestone, if maintained, might be an opportunity for malaria elimination in the near future for the two districts, especially adhering to the recommendation by the WHO [22] to target all villages with annual parasite index (API) of more than 5 cases per 1000 populations per annum. However, the new challenge is that the selection criteria of villages to be sprayed in each district by the Zimbabwe’s NMCP are not based on API, but are resource-based, leaving some villages with API of >5 % in Mutare and Mutasa Districts unsprayed. Hence, sporadic malaria outbreaks experienced in Mutare and Mutasa Districts in recent years have occurred in unsprayed villages with API of >5 % (Mberikunashe, personal communication), posing a serious new operational challenge to malaria control and elimination. Even with high IRS coverage of above 80 % for all villages with API of >5 %, the poor quality of spraying is a new challenge for malaria elimination in Mutare and Mutasa Districts. The poor quality of house spraying was revealed by WHO cone bioassay mortalities in *An. gambiaes.l.* of 34 % at a wall height <0.5 m and 100 % at a wall height of >1 m of the same structure, 24–48 h post spraying in Mutasa Districts [16].

**Table 1:** House spraying coverage and population protected in Mutare and Mutasa districts from 2009 to 2014

Years	Mutare		Mutasa		Manicaland		Zimbabwe	
	% cov.	% pop. prot.	% cov.	% pop. prot.	% cov.	% pop. prot.	% cov.	% pop. prot.
2009	98	99	99	86	90	86	85	80
2010	95	97	92	95	92	95	90	89
2011	89	100	86	93	90	96	93	92
2012	84	93	84	80	86	92	90	87
2013	80	95	87	85	83	83	91	90
2014	96	84	86	92	91	95	92	91

% cov. percentage coverage;  % pop. prot. percentage population protected

Moreso, part of the new challenge is with the sprayers themselves, where, in most instances, the standard compression sprayers and mode of application depend entirely on the ability and diligence of the spray operator to deliver the correct dose in the right location [31]. While the IRS programme in Mutare and Mutasa uses the WHO’s recommended compression sprayers, NMCP has not been able to consistently provide constant flow valve (CFV) for each sprayer over the years. The CFVs maintain a uniform application rate as the pressure in the tank falls and enhances overall efficiency of spraying [32].

To achieve the desired results in malaria control using IRS, Zimbabwe has been employing the WHO’s recommended insecticides. Dichloro-diphenyl-trichloro-ethane and BHC were used from 1945 to 1962, BHC independently in 1972 to 1973, DDT independently from 1974 to 1987, and deltamethrin and lambda-cyhalothrin from 1988 to 2000 [12]. Insecticides used for IRS from 2001 to 2013 are shown on Table 2 and it is clear that the NMCP used pyrethroids for 13 years consecutively in Mutare and Mutasa Districts. The choice of insecticide for use in IRS was primarily guided by cost and in 2014 the NMCP switched to organophosphates (pirimiphos-methyl) following the emergence of insecticide resistance in *An. funestus* in Mutare and Mutasa Districts [26, 30]. The lack of insecticide rotation suggests unavailability or non-use of insecticide resistance management plan which is a new challenge in achieving the malaria elimination goal.

**Table 2:** Insecticides, formulations and amounts used in Mutare and Mutasa districts for IRS from 2001 to 2014

Years	Insecticide	Class	Formulation	Amount used
2001	Lambda-cyhalothrin	Pyrethroids	10 WP	Not available
2002	Lambda-cyhalothrin	Pyrethroids	10 WP	Not available
2003	Lambda-cyhalothrin	Pyrethroids	10 WP	Not available
2004	Deltamethrin	Pyrethroids	5 WP	Not available
2005	Lambda-cyhalothrin	Pyrethroid	10 WP	Not available
2006	Lambda-cyhalothrin	Pyrethroid	10 WP	Not available
2007	Lambda-cyhalothrin	Pyrethroid	10 WP	Not available
2008	Lambda-cyhalothrin	Pyrethroid	10 WP	26,412 sachets
2009	Lambda-cyhalothrin	Pyrethroid	10 WP	27,564 sachets
2010	Lambda-cyhalothrin	Pyrethroid	5 WP	36,348 sachets
2011	Deltamethrin	Pyrethroid	5 WP	42,706 sachets
2012	Deltamethrin	Pyrethroid	5 WP	39,464 sachets
2013	Lambda-cyhalothrin	Pyrethroid	10 WP	36,643 sachets
2014	Pirimiphos-methyl	Organophosphate	300 CS	37,927 bottles

Traditionally, mosquito nets played a much lesser role than IRS until the initiation of LLIN campaigns under the universal coverage goal over the past few years. To fully implement the two vector control interventions, Zimbabwe had no clear guidance to inform provinces to balance the deployment strategies of LLINs and IRS following the WHO’s recommendation [33], especially the effectiveness of combining versus either IRS or LLINs alone, as well as the problem of introducing the second intervention as a means of compensating for the deficiencies in the implementation of the first.

Despite reports which showed more than 90 % mass distribution coverage of LLINs in Mutare and Mutasa Districts (Mberikunashe, unpublished data); net utilization data could not be easily accessed. However, utilization of mosquito nets amongst the population at risk in Manicaland Province was 47.5 % in 2012 (Zimbabwe Malaria Indicator Survey [ZMIS], unpublished data), 33.5 % short of the WHO’s 80 % coverage for impact. The low utilization of nets in the province may suggest equally low rate of utilization of the product in Mutare and Mutasa Districts. This poses a new challenge as the effectiveness of mosquito nets to combat malaria largely depends on their utilization by majority of people at risk.

## Resistance to anti-malarial medicines

The real need to intensify IRS arose when the first case of chloroquine resistance was confirmed from the Zambezi Valley, Zimbabwe in 1984 [34]. Chloroquine was then the first line anti-malarial medicine to treat uncomplicated malaria in Zimbabwe [35]. By 1989, chloroquine-resistant infections had been demonstrated in most parts of the endemic zones of the country, with varying types and levels of resistance [35–37].

Following confirmation of chloroquine resistance in several parts of Zimbabwe [34–37], chloroquine was replaced by a free combination of chloroquine and sulfadoxine-pyrimethamine (SP) as first-line of anti-malarial medicine in the early 2000s. Subsequent studies indicated rising failure of chloroquine and SP combination and these were replaced by artemisinin-based combination therapy (ACT) in 2004. The ACT anti-malarials were rolled out fully in 2007/8 and are currently in use (Zimbabwe Malaria Programme Review [ZMPR], unpublished data). Although Mutare and Mutasa Districts have not experienced major shortages of ACT and rapid diagnostic kits (RDT) over the past few years (Mberikunashe, unpublished data), the frequent introductions and replacements of anti-malarial medicines due to parasite resistance is a new challenge threatening the efforts towards malaria control and elimination in Zimbabwe. This situation is exacerbated when the same area experiences insecticide resistance in major malaria vectors.

## Status of insecticide resistance

Only four classes of insecticides are approved by the WHO to control malaria vector mosquitoes using house spraying [38]. These are pyrethroids, organochlorines, organophosphates and carbamates. At present, all the WHO-recommended LLINs [38] are treated with pyrethroids. The high dependence on pyrethroid-based malaria control has increased the selection pressure for insecticide resistance in malaria vectors. Even though insecticides have been used for a very long period in Zimbabwe, there are very few instances where resistance has been recorded [39]. Early reports of insecticide resistance in *An. arabiensis* appeared in the 1980s in Chiredzi District, south of Zimbabwe and showed BHC resistance [40]. Masendu et al. [9] and Munhenga et al. [39] reported resistance in *An. arabiensis* to DDT and permethrin from Gokwe District respectively, Zimbabwe. No further insecticide resistance was documented in Zimbabwe till recently, when pyrethroid and carbamate resistance was reported in *An. funestus* in Mutare and Mutasa Districts [26, 30]. Interestingly, the same studies showed that *An. funestus* populations were susceptible to both DDT (organochlorine) and pirimiphos-methyl (organophosphates).

While the emergence of insecticide resistance in *An. funestus* in Mutare and Mutasa Districts is a new challenge likely to reverse the gains made in malaria control, the lack of cross-resistance observed between pyrethroids and DDT, and carbamates and organophosphates is an opportunity for malaria control and elimination. However, following evidence of pyrethroid and carbamate resistance in *An. funestus* collected from Mutare and Mutasa Districts in 2014 [26, 30], Zimbabwe’s NMCP changed insecticide used for IRS from pyrethroids to pirimiphos-methyl 300 CS (organophosphate) in the same year. Although Kanyangarara et al. [41] showed that pirimiphos-methyl had a measurable impact on malaria incidence in Mutasa District, the new challenge with the use of pirimiphos-methyl 300 CS is that the cost is comparatively high and might be unsustainable to government and malaria stakeholders, leading to possible reversal of milestones gained in malaria control.

## Resting and biting behaviour of vectors *vis*-*a*-*vis* indoor house spraying and mosquito nets

The effectiveness of IRS and LLINs to prevent malaria transmission largely depends on resting and biting behaviours of the vectors. Indoor house spraying is effective against indoor resting mosquitoes, whereas LLINs control malaria vectors that bite indoors. Although several studies have shown the efficacy of IRS and LLINs in reducing malaria incidence in almost all settings [42–44], outdoor transmission is a new challenge to malaria control and elimination [18].

Studies in Gokwe and Binga Districts in Zimbabwe [14] showed that the principal vector *An. arabiensis* was partially exophilic, consequently, it might not be fully amenable to control by indoor application of residual insecticides, posing a new challenge to malaria control. Studies involving *An. gambiae* complex in Masakadza village, Gokwe South District in Zimbabwe [15] demonstrated predominantly exophilic tendencies of the complex, while its peak indoor biting activity occurred at 22:00 h, coinciding with times when some people would still be awake and out of mosquito nets. The observed biting times threatens malaria control and elimination using LLINs as a major vector control intervention. However, mosquito outdoor biting behaviour was not evaluated in this study.

Studies in Mutare and Mutasa Districts [16] established that 84 % of the *An. funestus* populations were endophilic, with a lower percentage exhibiting exophilic traits (16 %). Of those collected indoors, 90 % were collected on sprayable habitats (walls and roofs/ceiling) and 10 % on unsprayable surfaces (furniture and other household goods). Of those collected on sprayable surfaces, 56 % were collected on the roofs, with 44 % on the walls. For the past 5 years, the NMCP could not consistently spray roofs/ceiling owing to non-availability of extension lances to spray surfaces higher than 3.5 m from the ground level. Failure to spray roofs/ceiling on which the majority of mosquito species rest is a cause for concern and is a new challenge to malaria control and elimination programmes in Mutare and Mutasa Districts.

Sande et al. [17] reported trapping *An. funestus* populations and *An. gambiae s.l.* more abundantly indoors (68 %) than outdoors (32 %) using Centers for Disease Control and Prevention (CDC) traps, suggesting that malaria could be interrupted by LLINs if the strategy is used by the majority of residents in Mutare and Mutasa Districts. However, the observed variable nocturnal host-seeking behaviour of *An. funestus* in Mutare and Mutasa Districts, with two peaks during the night; between 22:00–23:00 and 02:00–04:00 h is a new challenge to malaria control and elimination. Both peaks suggest that malaria transmission might be maintained despite net ownership and use as this was a period when probably a fairly small proportion of the rural population might not have gone to bed yet or might have got out of bed already for early morning household chores.

## Conclusion

Opportunities and critical new challenges to the ambitious goal of malaria elimination exist in Mutare and Mutasa Districts of Manicaland Province in Zimbabwe. The predominant endophilic behaviour and high indoor blood seeking traits of *An. funestus*, lack of cross resistance between pyrethroids and DDT, carbamates and organophosphates, as well as scaled-up malaria control interventions, especially high house-spray coverage or LLIN distribution, the existing political will, and the Zimbabwe NMCP’s commitment to E8 agenda create prospects for malaria elimination in Mutare and Mutasa Districts in the near future. However, realising the opportunities to achieve malaria elimination goal does not provide justification for ignorance to critical new challenges which have the potential to seriously retard progressing towards regional ambitious goal of malaria elimination. The emergence of resistance to anti-malarial medicines and insecticides, failure to spray all villages with an API of >5 %, poor spray quality in some instances, unavailability of clear guidelines on the deployment of IRS and LLINs, the use of alternatives and possible more costly insecticide in IRS to maintain the required level of vector control interventions, as well as the resurgence of one of the most efficient malaria vectors, *An. funestus*, non-spraying of roofs/ceiling where majority of mosquitoes prefer to rest, and possible outdoor transmission, are the new challenges threatening the milestones gained towards malaria control and elimination in Mutare and Mutasa Districts.

Evidence presented in this review suggests that selection of malaria intervention strategies in Mutare and Mutasa Districts, especially anti-malarial medicines, insecticides for IRS and use of pyrethroid-based LLINs should be based on susceptibility status to anti-malarials and insecticides, as well as resting and biting behaviour of the vector mosquitoes. These aspects are important to achieve global health agenda for malaria elimination. The NMCP and stakeholders should devise an insecticide resistance management plan as part of their vector control activities. Systematic monitoring of resistance to anti-malarial medicines and insecticides, and studies on malaria vector species composition, resting and biting behaviour has to be strengthened. All results on entomological monitoring surveys conducted in any region of Zimbabwe have to be rapidly and widely disseminated to pertinent government health staff, WHO and other relevant stakeholders in the field of malaria prevention, control and elimination. It is important to closely monitor outdoor transmission of malaria and the selection of malaria intervention strategies and their implementation in Mutare and Mutasa Districts in Zimbabwe should always be evidence-based.

## Abbreviations

ACT: artemesinin-based combination therapy
API: annual parasite index
BHC: benzene hexachloride
CDC: Centers for Disease Control and Prevention
CFV: constant flow valve
CS: capsule suspension
DDT: dichloro-diphenyl-trichloro-ethane
E8: elimination eight
IRS: indoor residual spraying
LLINs: long-lasting insecticidal nets
MGD: Millennium Development Goal
NIHR: National Institute of Health Research
NMCP: National Malaria Control Programme
RBM: Roll Back Malaria
RDT: rapid diagnostic kits
SP: sulfadoxine–pyrimethamine
ZDHIS2: Zimbabwe District Health Information System two
ZMIS: Zimbabwe Malaria Indicator Survey
MPR: Zimbabwe Malaria Programme Review
